# Classification of quantum-spin-hall topological phase in 2D photonic continuous media using electromagnetic parameters

**DOI:** 10.1515/nanoph-2025-0451

**Published:** 2025-10-23

**Authors:** Xin-Tao He, Shuo-Shi Zhang, Xiao-Dong Chen, Jian-Wen Dong

**Affiliations:** School of Physics & State Key Laboratory of Optoelectronic Materials and Technologies, 26469 Sun Yat-Sen University, Guangzhou, 510275, China

**Keywords:** topological photonics, continuous media, electromagnetic parameters, phase map

## Abstract

Refractive index is a fundamental electromagnetic (EM) parameter that can describe photonic continuous media (PCM) traditionally as either transparency or opacity. Recently, topological theory offers a new set of phases to characterize PCM as either trivial or nontrivial, by using topological invariant which are not direct to EM parameters. As all of the optical properties in PCM should be related to EM parameters, we formulate a topological index based on EM parameters and establish its phase map in this work. The map can analytically describe the deterministic condition for a topologically nontrivial phase. Our findings indicate that the topology of 2D bi-anisotropic PCM is determined by the sign of the topological index. Another EM parameter of pseudo surface impedance is also introduced for the opaque regions of PCM, showing the topological opacity has a full range of impedance values ranging from negative to positive, while the trivial case only has either negative or positive impedance. The simulation results show that an interface between two opacities with differing index signs can support robustly optical propagation of topological edge states. The proposal of EM-parameter method reveals a deep understanding on topological properties of PCM, and will enrich the topological theory in photonic systems.

## 1 Introduction

Topology, a concept originating from mathematics, has attracted widespread attention in the physics world, from condensed matter to classical waves [1], [2]. The rapid developments in topological photonics [3], [4], [5], [6], [7] have created an advanced platform at both microwave and optical frequencies for flexibly controlling various types of topological phases, such as quantum anomalous/spin/valley Hall effects [8], [9], [10], [11], [12], [13], [14], [15], [16], [17], [18], [19], [20], [21] and 1D/2D Zak phases [22], [23], [24], [25], [26], [27], [28], [29], [30], [31]. Furthermore, topological photonics give a new paradigm to design on-chip devices, especially promising for nanophotonics and integrated optics [15], [32], [33], [34], [35]. These intriguing features in principle are derived from the topological band theory, which can theoretically predict nontrivial evolution of band structures and protections of topological states in the *k* space [36]. For photonic systems, a general method to mimic the effective model of topological band theory is the exploration of photonic periodic lattice (PPL). As one of the common cases, photonic crystals support Bloch waves in its building blocks [7], [37], and some of the eigenmodes around high-symmetry *k* points can be approximately described by the effective Hamiltonian. Based on the band-eigenfield method, one can calculate Berry curvature and topological invariant to characterize topological phases as either non-trivial or trivial, by exploring the evolution of photonic bands and eigenfields in *k* space.

Photonic continuous media (PCM) are a class of optical homogeneous materials, such as metamaterials which are homogenized as effective media. As well known, most of metamaterials or artificial PCM can be described by a set of electromagnetic (EM) parameters [38], [39], i.e. permittivity *ε* and permeability *μ*. Based on the sign of *ε* and *μ*, such PCM have achieved lots of exotic EM responses which are difficult to be realized by natural existence of optical materials, including ultrahigh-index permittivity (*ε* >> 1, *μ* > 0), negative index (*ε* < 0, *μ* < 0), magnetic mirrors (*ε* > 0, *μ* < 0) and zero refractive index ( $n=\sqrt{\varepsilon \mu }\approx 0$ ). Despite of the absence of Bloch-wave mechanism, topologically non-trivial phases also extend to PCM with certain material dispersion, which is analogous to the topological bands of PPL. In this way, PCM could maintain variety of topological features. One-way waveguide were realized in an interface between a 2D magnetized plasma and an ordinary reflector [40], [41], which shows an evidence for the topological edge states in PCM. Later, an efficient method was developed to calculate the topological invariant of dispersive magnetized plasma [42] and dual-symmetric gyrotropic-materials [43], which give a direct characterization of quantum-Hall topological phase. It was also found that 3D PCM with chiral response could be associated with topological phase and Weyl point [44], [45], [46], [47], [48], as well as be extended to intrinsic topological hinge states [49]. The topological features of zero-refractive-index materials were analyzed by the concept of direction-dependent refractive index [50] and are recently related to bulk-spatiotemporal vortex correspondence [51]. The retrieval of topologically non-trivial phases in these works is mostly derived from band-eigenfield theory. However, it is not straightforward for PCM, as all of the optical responses should be associated with their EM parameters. This issue inspires us to seek a new approach to bridge the gap between topological invariant and EM parameters, which will be of high significance in optical sciences and metamaterial applications.

**Figure 1:**
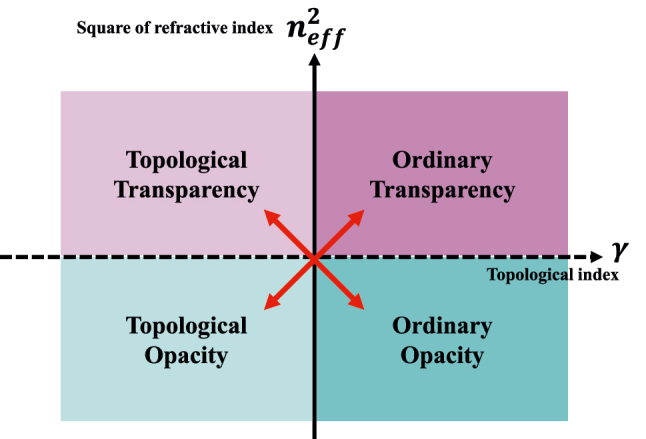
Topological phase map of photonic continuous media (PCM), which is divided into four quadrants, i.e. ordinary transparency, topological transparency, topological opacity and ordinary opacity. Red arrows indicate two types of topological transition. The topological index indicates the in-plane EM constitutive difference between diagonal and off-diagonal elements.

In this work, as the consequences that the nonzero topological invariant is derived from the singularity of Berry curvature, we propose an EM-parameter method that defines a topological index related to the condition of the singularity. Such defined topological index indicates the in-plane EM constitutive difference between diagonal and off-diagonal elements. Based on refractive index and topological index, a topological phase map (see Figure 1) is constructed to characterize the quantum-spin-Hall topological phase in 2D PCM, which is determined by the sign of topological index. An example of PCM with Lorentz-like dispersion is given to show that the evolution of topological index is well consistent with band theory. An interface between two opacities of different index sign gives rise to gapless edge states against sharp bending. Such bulk-edge correspondence is also described by pseudo surface impedance of complete gap. Compared with previous works of PCM on band-eigenfield method, the proposal of EM-parameter method reveals a deep understanding on topological properties of PCM beyond topological bands, and simplifies the design of topological metamaterial in some manner.

## 2 Results and discussion

### 2.1 Theoretical model of bi-anisotropic PCM

In this work, we focus on a photonic continuous medium (PCM) with bi-anisotropic (BI) response. Such PCM has the following constructive relations,

(1) $$\overset{⇀}{D}=\varepsilon_{0}{\overset{↔}{\varepsilon }}_{r}\overset{⇀}{E}+\left(\overset{↔}{\chi }/c\right)\overset{⇀}{H},\overset{⇀}{B}=\mu_{0}{\overset{↔}{\mu }}_{r}\overset{⇀}{H}+\left(\overset{↔}{\chi }/c\right)\overset{⇀}{E},$$

where

(2) $$\begin{array}{ll} {\overset{↔}{\varepsilon }}_{r} & =\left(\begin{array}{ccc} \varepsilon_{p} & 0 & 0\\ 0 & \varepsilon_{p} & 0\\ 0 & 0 & \varepsilon_{z} \end{array}\right),{\overset{↔}{\mu }}_{r}=\left(\begin{array}{ccc} \mu_{p} & 0 & 0\\ 0 & \mu_{p} & 0\\ 0 & 0 & \mu_{z} \end{array}\right),\\ \overset{↔}{\chi } & =\left(\begin{array}{ccc} 0 & -i\xi & \\ i\xi & 0 & \\ & & 0 \end{array}\right), \end{array}$$

are the relative permittivity, relative permeability and bi-anisotropic tensors, respectively. The subscript *p* and *z* represent in-plane (perpendicular to x-y plane) and out-of-plane (vertical to x-y plane) components. For harmonic waves in passive system, the Maxwell equations with bi-anisotropic response can be written as,

(3) $$\nabla \times \overset{⇀}{E}=i\omega \left[\mu_{0}{\overset{↔}{\mu }}_{r}\overset{⇀}{H}+\left(\overset{↔}{\chi }/c\right)\overset{⇀}{E}\right],$$

(4) $$\nabla \times \overset{⇀}{H}=-i\omega \left[\varepsilon_{0}{\overset{↔}{\varepsilon }}_{r}\overset{⇀}{E}+\left(\overset{↔}{\chi }/c\right)\overset{⇀}{H}\right],$$

where *ε*_0_, *μ*_0_ and *c* are the permittivity, permeability and velocity of light in vacuum, respectively. According to Eqs. (3) and (4), we can see the interaction of the electric fields and magnetic fields due to bi-anisotropic response.

In general, different optical materials respond differently for electric field and magnetic field, i.e., EM-dual symmetry breaking. For example, the ratio of medium 1 (*ρ*_1_ = *ɛ*_*p*1_/*μ*_*p*1_ = *ɛ*_*z*1_/*μ*_*z*1_) is quite different from that of medium 2 (*ρ*_2_ = *ɛ*_*p*2_/*μ*_*p*2_ = *ɛ*_*z*2_/*μ*_*z*2_). In order to construct a pair of pseudo spin degenerating, one of the solutions is to retrieve electromagnetic-dual parameter that ensures the ratio *ρ* as a constant in the whole space. Thus, the Maxwell equations can be divided into two non-relativistic equations for two sets of decoupled vectors ${\overset{⇀}{\psi }}^{\pm }={\left(\begin{array}{ccc} \sqrt{\varepsilon_{0}\rho }E_{x}\mp \sqrt{\mu_{0}}H_{x} & \sqrt{\varepsilon_{0}\rho }E_{y}\mp \sqrt{\mu_{0}}H_{y} & \sqrt{\varepsilon_{0}\rho }E_{z}\pm \sqrt{\mu_{0}}H_{z} \end{array}\right)}^{T}$ , where the superscript + (−) represents the spin-up (spin-down) eigenstate for in-phase (anti-phase) field pattern between *E*_z_ and *H*_z_. The detailed derivation can be seen in Appendix A of the Supplementary Materials. Note that all of ‘spin’ in this work represent pseudo spin.

### 2.2 Bulk topology of PCM in momentum space

**Figure 2:**
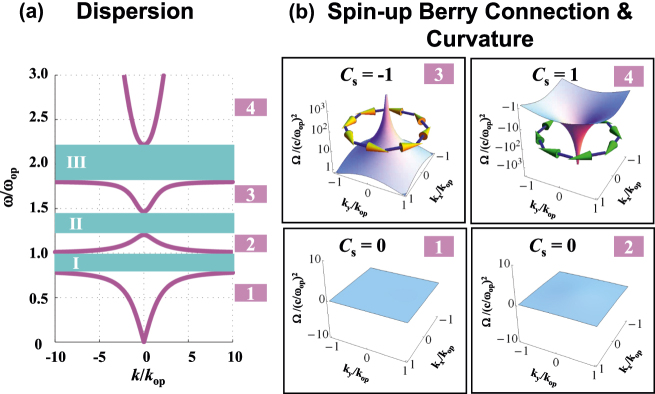
Topological phase in a bi-anisotropic (BI) PCM with electromagnetic (EM) duality $\overset{↔}{\varepsilon }=\overset{↔}{\mu }=\mathrm{diag}\left(\varepsilon_{p},\varepsilon_{p},\varepsilon_{z}\right)$ . (a) Dispersion relation of electromagnetic-dual bi-anisotropic PCM. *ω*_*op*_ is the in-plane oscillation frequency, and *k*_op_ = *ω*_op_/*c* is the wavevector related to *ω*_op_. The pink lines represent the band dispersions of two spin-degenerate bulk modes (referred as bands 1–4), while there are three cyan gaps I-III separated by these bands. (b) Spin-up Berry curvature Ω^+^ and schematic view of spin-up Berry connection ${\overset{⇀}{A}}^{+}$ for bands 1–4. For band 3 (4), we can clearly observe the singular peak (dip) of Berry curvature at the center of *k* space, which is accompanied with anticlockwise (clockwise) vortex of Berry connection.

For simplicity, we will discuss a special case of EM duality with *ρ* = 1 [ ${\overset{↔}{\varepsilon }}_{r}={\overset{↔}{\mu }}_{r}=\mathrm{diag}\left(\varepsilon_{p},\varepsilon_{p},\varepsilon_{z}\right)$ ]. With a simple deduction, the Maxwell equations can be generalized to be Schrödinger-like formation ${\overset{↔}{H}}^{\pm }{\overset{⇀}{\psi }}^{\pm }=\frac{\omega }{c}{\overset{⇀}{\psi }}^{\pm }$ (see Appendix B of the Supplementary Materials). The characteristic matrix ${\overset{↔}{H}}^{\pm }$ is the effective Hamiltonian of PCM, which is similar to the role of Hamiltonian in quantum system. The eigenfields in PCM can be simplified as plane-wave form that ${\overset{⇀}{\psi }}^{\pm }={\overset{⇀}{\varphi }}^{\pm }\exp\left(i\overset{⇀}{k}\cdot \overset{⇀}{r}\right)$ , where ${\overset{⇀}{\varphi }}^{\pm }$ is the complex amplitude of the spin-polarized states. After solving the eigenvalues of effective Hamiltonian, the bulk dispersion is given as,

(5) $$k_{x}^{2}+k_{y}^{2}=k^{2}={\left(\frac{\omega }{c}n_{\mathrm{eff}}\right)}^{2},$$

where $n_{\mathrm{eff}}=\sqrt{\varepsilon_{z}\left(\varepsilon_{p}^{2}-\xi^{2}\right)/\varepsilon_{p}}$ is the effective refractive index. Equation (5) is the characteristic equation for both spin–up and–down modes, since they degenerate simultaneously in the bulk. Consider the BI PCM with Lorentz-like dispersion as the following form,

(6) $$\begin{array}{ll} \varepsilon_{p} & =\mu_{p}=1+\frac{\omega_{A}^{2}}{\omega_{op}^{2}-\omega^{2}},\xi =\frac{\omega_{B}\omega }{\omega_{ok}^{2}-\omega^{2}},\\ \varepsilon_{z} & =\mu_{z}=1+\frac{\omega_{C}^{2}}{\omega_{oz}^{2}-\omega^{2}}, \end{array}$$

where *ω*_*op*_, *ω*_*ok*_ = *ω*_*op*_, *ω*_*oz*_ = 0.8*ω*_*op*_, are the oscillation frequencies for different components, while *ω*_*A*_ = 1.5*ω*_*op*_, *ω*_*B*_ = 0.75*ω*_*op*_, *ω*_*C*_ = 0.9*ω*_*op*_ represent the resonance strength, respectively. Here all parameters are normalized by the in-plane oscillation frequency *ω*_*op*_. Applying Eq. (6) to Eq. (5), we have analytic solution of the dispersion relation as shown in Figure 2a. The pink lines represent the band dispersions of two spin-degenerate modes. To subsequent discussion we refer to these four bands as bands 1–4, respectively. Separated by these bulk bands, there are three cyan gaps as the following frequency intervals: *ω*_*oz*_ < *ω* < *ω*_*op*_ for gap I, $\sqrt{\omega_{oz}^{2}+\omega_{C}^{2}}<\omega <\left[-\omega_{B}+\sqrt{\omega_{B}^{2}+4\left(\omega_{op}^{2}+\omega_{A}^{2}\right)}\right]/2$ for gap II and $\sqrt{\omega_{op}^{2}+\omega_{A}^{2}}<\omega <\left[\omega_{B}+\sqrt{\omega_{B}^{2}+4\left(\omega_{op}^{2}+\omega_{A}^{2}\right)}\right]/2$ for gap III.

To study the topological properties of those bulk bands and gaps, we should attain the Berry information, including the Berry connection ${\overset{⇀}{A}}^{\pm }$ , Berry curvature Ω^±^ and the corresponding spin Chern number *C*_*s*_. By using the generalized method (see Appendix C of the Supplementary Materials) and nonlocal approximation (see Appendix D of the Supplementary Materials), the Berry connection ${\overset{⇀}{A}}^{\pm }$ are obtained as follow,

(7) A⇀±=Reiφ⇀±∗⋅∂∂ωωM↔±⋅∇kφ⇀±φ⇀±∗⋅∂∂ωωM↔±⋅φ⇀±=Ax±k⇀x+Ay±k⇀y,

where $\nabla_{k}=\frac{\partial }{\partial k_{x}}{\overset{⇀}{k}}_{x}+\frac{\partial }{\partial k_{y}}{\overset{⇀}{k}}_{y}$ is the Laplace operator in 2D momentum space, and thus the Berry curvature $\Omega^{\pm }=\nabla_{k}\times {\overset{⇀}{A}}^{\pm }$ in PCM can be calculated analytically. Based on the integration of Berry curvatures over entire *k* space, we have a quantized invariant to define the topology of spin–up and–down channels,

(8) $$C^{\pm }=\frac{1}{2\pi }\iint \Omega^{\pm }dk_{x}dk_{y}.$$

The net Chern number always vanishes (i.e. *C* ^+^ + *C* ^−^ = 0) due to time-reversal invariance. The topological phase of the overall system can be characterized by another invariant, i.e. spin Chern number $C_{s}=\left(C^{+}-C^{-}\right)/2=C^{+}$ . Note that the smooth distribution of Berry curvature satisfies Stokes’ theorem: $C_{s}=\frac{1}{2\pi }\iint \Omega^{+}dk_{x}dk_{y}=\frac{1}{2\pi }\oint_{k=\infty }{\overset{⇀}{A}}^{+}\cdot d\overset{⇀}{l}$ [bands 1 and 2 in Figure 2b] and thus the surface integral of Berry flux can be replaced by the line integral of Berry gauge field over the *k*-space boundary (*k* = *∞*). The well-defined effective Hamiltonian ensures the Berry connection to be vanish at *k* = *∞*, so that the line integral of equifrequency contour of infinite wavevector attains $C_{s}=\frac{1}{2\pi }\oint_{k=\infty }{\overset{⇀}{A}}^{+}\cdot d\overset{⇀}{l}\equiv 0$ . To obtain nonzero *C*_*s*_, the singularity of Berry curvature should be found. In band 3 (4) of Figure 2b, we can clearly observe singular peak of spin-up Berry curvature with anticlockwise (clockwise) vortex of Berry connection. The singular vortex indicates a source of Berry gauge field in accordance with the fact that *C*_*s*_ is −1 (+1). In these two cases, the Strokes’ theorem can be recovered by adding a summation of line integrals in the vicinity of singular *k* points, i.e. $C_{s}=\frac{1}{2\pi }\oint_{k=\infty }{\overset{⇀}{A}}^{+}\cdot d\overset{⇀}{l}-\frac{1}{2\pi }\sum_{k_{i}}\oint_{R^{+}}{\overset{⇀}{A}}^{+}\cdot d\overset{⇀}{l}$ , where *R* ^+^ is a circle in the vicinity of singular ${\overset{⇀}{k}}_{i}$ point. The summation term provides nonzero value of spin Chern number and thus leads to a topological phase transition. In other words, to guarantee the existence of topological nontrivial phase, it is more significant to explore the singularities of Berry curvature.

### 2.3 Topological phase transition characterized by electromagnetic parameters

**Figure 3:**
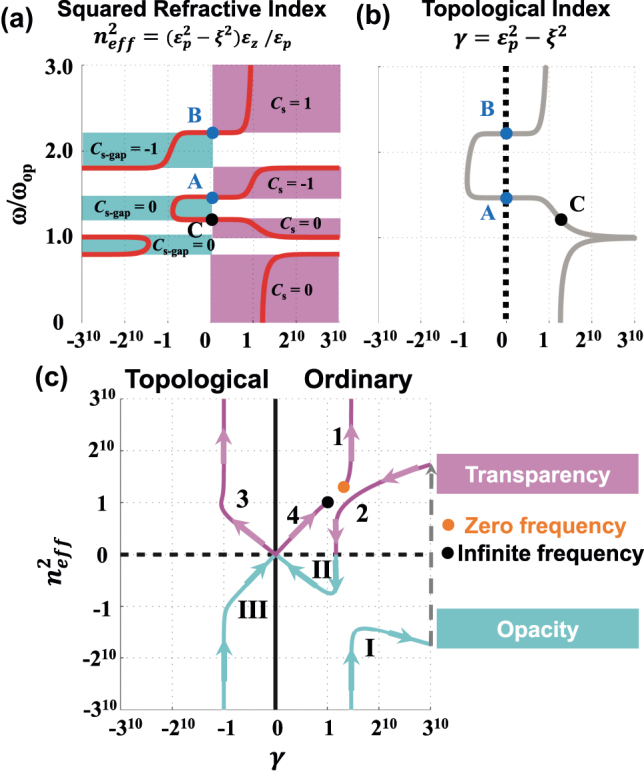
Characterization of topological phase transition based on electromagnetic parameters. (a) The square of effective refractive index (SERI) $n_{\text{eff}}^{2}=\varepsilon_{z}\left(\varepsilon_{p}^{2}-\xi^{2}\right)/\varepsilon_{p}$ as a function of frequency. (b) Topological index $\gamma =\varepsilon_{p}^{2}-\xi^{2}$ as a function of frequency. Two zero-topological-index points ( $\varepsilon_{p}^{2}-\xi^{2}=0$ ) are highlighted as blue dots. (c) Evolution of topological phase map as the operation frequency increases continuously. The material response and numerals labels are identical to Figure 2a.

In fact, regardless of band theory, the optical properties of PCM should be related to its EM parameters. This issue inspires us to bridge the gap between topological phase and EM parameters. We start with a fundamental parameter, i.e. the square of effective refractive index (SERI) $n_{\mathrm{eff}}^{2}=\varepsilon_{z}\left(\varepsilon_{p}^{2}-\xi^{2}\right)/\varepsilon_{p}$ . Figure 3a show the SERI of bi-anisotropic PCM as a function of frequency. The transport properties are traditionally determined by the sign of SERI. For $n_{\mathrm{eff}}^{2}>0$ (pink bands), the transparent waves propagate in the bulk media freely, while the opaque modes localize in the surface and exponentially decay into the bulk when $n_{\mathrm{eff}}^{2}<0$ (cyan gaps). As SERI cannot characterize the topology of PCM, we should seek a new index that can describe the relationship between topological phase and EM parameters.

As stated above, the transition of topological phase is related to the singularity of Berry curvature. The expression of Berry curvature can be analytically written as

(9) $$\begin{array}{ll} \Omega^{\pm } & =\mp 2\left[\left(\xi +\omega \partial_{\omega }\xi \right)\left(\varepsilon_{p}^{2}+\xi^{2}\right)-2\xi \varepsilon_{p}\left(\varepsilon_{p}+\omega \partial_{\omega }\varepsilon_{p}\right)\right]\\ & \;/\left[W_{0}k_{0}^{2}{\left(\varepsilon_{p}^{2}-\xi^{2}\right)}^{2}\right], \end{array}$$

where *W*_0_ is proportional to the time-averaged energy density that *W*_0_ > 0. To observe the distribution of Berry curvature and retrieve topologically nontrivial phase, all of the following discussions focus on nonzero BI coefficient. According to Equation (9), the Berry curvatures go to infinity when the term $\varepsilon_{p}^{2}-\xi^{2}$ approaches to zero and thus leads to a transition of topological phase. In other words, we can use such index $\gamma =\varepsilon_{p}^{2}-\xi^{2}$ to characterize the topology of PCM. The physical meaning of the defined topological index *γ* is the in-plane constitutive difference between diagonal element *ε*_*p*_ and off-diagonal element *ξ*. Figure 3b plots the topological index $\gamma =\varepsilon_{p}^{2}-\xi^{2}$ as a function of operation frequency. Two special points ( $\varepsilon_{p}^{2}-\xi^{2}=0$ ) are highlighted as blue dots. They guarantee the existence of the singularity of Berry curvature which leads to topological transition. Below point A, including bands 1–2 and gaps I-II, the material attributes to trivial topology with topological index $\varepsilon_{p}^{2}-\xi^{2}>0$ . At the intersection between gap II and band 3, the topological index experiences from positive to negative value. The change of sign of topological index leads to the singular structure of Berry curvature and provides a topological transition from trivial to nontrivial phase. Therefore, the region with negative topological index carries topologically nontrivial properties. Similarly, another topological transition can be also observed around point B. Note that the topological transition points ( $\varepsilon_{p}^{2}-\xi^{2}=0$ ) will inevitably appear at the origin of phase map due to $n_{\mathrm{eff}}^{2}=\varepsilon_{z}\left(\varepsilon_{p}^{2}-\xi^{2}\right)/\varepsilon_{p}$ . In other words, the topological phase transition occurs at the effective zero-refractive-index point. But we should note that it is not all of the zero-refractive-index point attribute to topological phase transition, i.e. point C. The above discussions don’t rely on a specific dispersion. According to the derivation from Equation (1) to Equation (9), we can hold the same conclusion of Equation (9) and derive the index *γ*, regardless of the form of dispersion.

On the other hand, we can apply the solutions of eigenfields [Eq. (S12) in the Supplementary Materials] to reveal the underlaying physics of topological index *γ*. Consider the eigenfields propagating along *y* axis (*k*_*x*_ = 0), and the complex amplitude can be simplified as,

(10) $${\overset{⇀}{\varphi }}^{\pm }={\left[\begin{array}{ccc} \mp \frac{k_{y}\varepsilon_{p}}{k_{0}\left(\varepsilon_{p}^{2}-\xi^{2}\right)} & -i\frac{k_{y}\xi }{k_{0}\left(\varepsilon_{p}^{2}-\xi^{2}\right)} & 1 \end{array}\right]}^{T}.$$

For *ξ* = 0, the eigenfields propagating along *y* axis are linearly polarized in *x-z* plane. For *ξ* ≠ 0, the eigenfields possess longitudinal component ( ${\overset{⇀}{\varphi }}_{y}^{\pm }\neq 0$ ) that generate ‘chiral’ profile in *x-y* plane, and underlaying physics can be discussed as two cases: (i) the positive topological index $\gamma =\varepsilon_{p}^{2}-\xi^{2}>0$ indicates the in-plane eigenfields are dominant for transverse component ( $\left|{\overset{⇀}{\varphi }}_{x}^{\pm }\right|>\left|{\overset{⇀}{\varphi }}_{y}^{\pm }\right|$ ), which is similar to *ξ* = 0 case and should be classified as topologically trivial phase. (ii) the negative topological index $\gamma =\varepsilon_{p}^{2}-\xi^{2}<0$ indicates the in-plane eigenfields are dominant for longitudinal component ( $\left|{\overset{⇀}{\varphi }}_{x}^{\pm }\right|<\left|{\overset{⇀}{\varphi }}_{y}^{\pm }\right|$ ), which can be classified as non-trivial phase.

A topological phase map is defined to combine the information of SERI and topological index, as shown in Figure 1. The phase map can be divided into four quadrants, i.e. ordinary transparency, topological transparency, topological opacity and ordinary opacity. There are two types of topological transition highlighting by red arrows. As SERI $n_{\mathrm{eff}}^{2}=\varepsilon_{z}\left(\varepsilon_{p}^{2}-\xi^{2}\right)/\varepsilon_{p}$ include the term of topological index $\varepsilon_{p}^{2}-\xi^{2}$ , the topological transition points ( $\varepsilon_{p}^{2}-\xi^{2}=0$ ) will inevitably appear at the origin of phase map. In other words, the transition of topology will always be accompanied by transparent-opaque transition. Figure 3c gives the evolution of phase map in bi-anisotropic PCM, as the operation frequency increases continuously. The material response and numerals labels are identical to Figure 2a. The medium first behaves as ordinary transparency (band 1) and then turns to be ordinary opacity (gap I) with a resonance of *ɛ*_*z*_. As the increasing of operation frequency, the optical responses jump back to the first quadrant (band 2) and passes through the horizontal axis without resonance. We should note that all above trajectories locate at the topologically trivial semi-space. And then, two types of topological transition are found near the origin of coordinate: the first one is from ordinary opacity to topological transparency [i.e. point A in Figure 3b], while the second one is between topological opacity and ordinary transparency [i.e. point B in Figure 3b].

Here, we discuss the expandability of the proposal EM-parameter method, which applies the sign of in-plane constitutive difference $\varepsilon_{p}^{2}-\xi^{2}$ to characterize the topological phase. For *ρ* ≠ 1, the definition of topological index can be generalized as $\gamma ={\left(\varepsilon_{p}/\sqrt{\rho }\right)}^{2}-\xi^{2}$ and the findings of Figure 3 retain validity. Furthermore, although this work focuses on quantum-spin-Hall phase of bi-anisotropic media, the EM-parameter method can be generalized to quantum-Hall topology of gyroelectric or gyromagnetic PCM, since they have the same form of master equations to bi-anisotropic PCM (see Appendix E of the Supplementary Materials). Therefore, our method is also compatible to analyze the quantum-Hall topology of magnetized plasma [41], [42] and the photonic analogues of the Haldane model [52]. Except for PCM, the lattice models (e.g. waveguide arrays, ring resonators and synthetic lattices) may develop a similar method to characterize the topological phases [13], [53], [54], if one can construct some effective parameters (by replacement of photonic dispersion/band) to totally describe the optical responses of the system.

### 2.4 Relationship between pseudo surface impedance and topological phase

**Figure 4:**
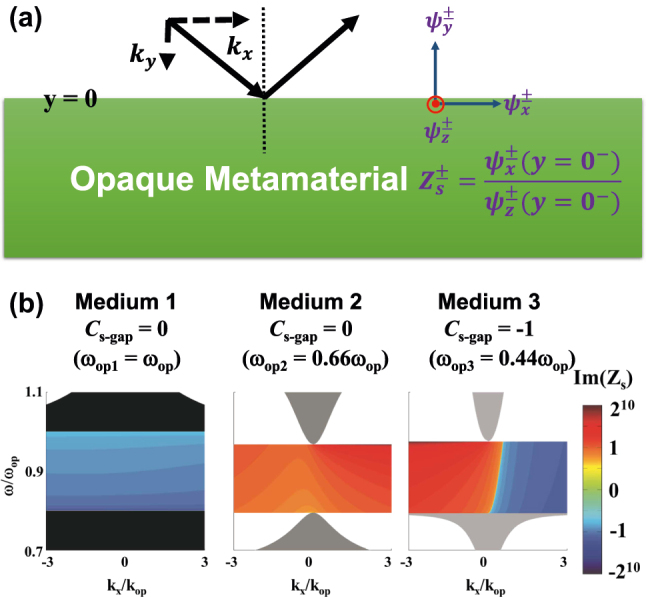
Relationship between pseudo surface impedance and topological phase. (a) Definition of pseudo surface impedance as the ratio of the *x*-direction spin wave to the *z*-direction spin wave. (b) Imaginary part of surface impedance for spin-up states in three different PCM, as the same EM parameters to Figure 2 except for normalized in-plane oscillation frequency. For simplicity, the pseudo surface impedances are only given in the opaque regions of each medium.

On the other hand, the surface impedance is related to the existence of interface states. Inspired by this method, we introduce the concept of surface impedance into the bi-anisotropic PCM. Figure 4a shows the definition of pseudo surface impedance on the downward side of the boundary as the ratio of the *x*-direction spin wave to the *z*-direction spin wave, yielding $Z_{s}^{\pm }=\psi_{x}^{\pm }\left(y=0^{-}\right)/\psi_{z}^{\pm }\left(y=0^{-}\right)$ , where *y* = 0 defines the boundary. Based on Eq. (S12) of Supplementary Materials, we can analytically obtain the pseudo surface impedance in theory. Here, we only focus on the pseudo surface impedances in the opaque regions, whose real part vanish in the complete gap. The imaginary part of pseudo surface impedances for spin-up states are presented in Figure 4b. These three different PCMs have the same EM parameters to Figure 2 except for normalized in-plane oscillation frequency. Medium 1 preform as a topologically trivial opacity in the gap region with negative impedance, while medium 2 possesses positive impedance in the topologically trivial opaque region. The topologically non-trivial opacity (medium 3) experiences full-range impedance from positive infinity to negative infinity. To guarantee the existence of an interface state, one should retrieve matched impedances on each side of interface, i.e. *Z*_*s*1_ + *Z*_*s*2_ = 0. For example, a traditional method is to form an interface between positive-impedance opacity and negative-impedance opacity. However, some certain frequencies cannot verify the impedance-matched condition, such that there is lack of surface waves. The topologically nontrivial gap, with full-range impedance from positive infinity to negative infinity, gives a robust mechanism to match another impedance of trivial gap. This is another demonstration why the gapless dispersion of surface waves can be formed at the interface between nontrivial gap (opacity) and trivial gap (opacity).

### 2.5 Optical propagation of topological edge states in PCM

**Figure 5:**
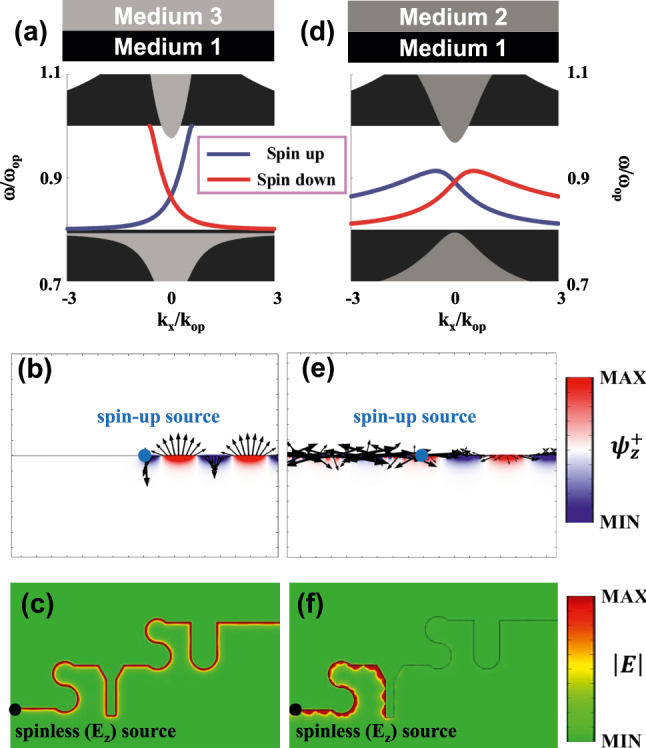
Optical propagation in topologically non-trivial (left) and trivial (right) interfaces. (a) Edge dispersion at an interface between an *C*_*s*_ = 0 ordinary opacity (medium 1) and a *C*_*s*_ = −1 topological opacity (medium 3). (b) Rightward unidirectional couple excited by a spin-up point source (blue dot). The black arrows represent the in-plane components (*ψ*_*x*_, *ψ*_*y*_) of edge states. (c) Robust propagation of topological edge state against the ‘SYSU’ shaped bending, by using a spinless *E*_z_-polarization source (black dot). (d) Edge dispersion supported by an interface between two ordinary opacities (medium 1 and medium 2). (e) and (f) Same numerical setup to the left panel, except for topologically trivial interface.

Next, we will focus on the propagation of edge states forming by two diverse opacities. To get a better understanding of spinful edge states, we construct a one-dimensional interface along *x* axis formed between two semi-infinite materials. The edge dispersion for spin-up and -down can be presented as follows,

(11) $$\begin{array}{ll} & \pm k_{x}\frac{\xi_{1}}{\varepsilon_{p1}^{2}-\xi_{1}^{2}}+\frac{\varepsilon_{p1}\sqrt{k_{x}^{2}-k_{0}^{2}\left(\varepsilon_{p1}^{2}-\xi_{1}^{2}\right)\varepsilon_{z1}/\varepsilon_{p1}}}{\varepsilon_{p1}^{2}-\xi_{1}^{2}}\\ & \;=\pm k_{x}\frac{\xi_{2}}{\varepsilon_{p2}^{2}-\xi_{2}^{2}}-\frac{\varepsilon_{p2}\sqrt{k_{x}^{2}-k_{0}^{2}\left(\varepsilon_{p2}^{2}-\xi_{2}^{2}\right)\varepsilon_{z2}/\varepsilon_{p2}}}{\varepsilon_{p2}^{2}-\xi_{2}^{2}} \end{array}$$

where the subscripts 1 and 2 represent the materials on each side of interface. ‘+’ and ‘-’ attribute to spin-up and spin-down cases, indicating they have opposite propagation direction for each other. After solving Eq. (11), we can obtain the edge dispersion at an interface between an *C*_*s*_ = 0 ordinary opacity (medium 1) and a *C*_*s*_ = −1 topological opacity (medium 3) in Figure 5a. As the time-reversal partners, the dispersions for spin-up (blue) and spin-down (red) edge states are symmetrical with respect to the plane of *k*_x_ = 0, and gaplessly cross over the complete gap. Consequently, the rightward unidirectional propagation can be excited by a spin-up point source [blue vortex in Figure 5b], as expected from the bulk topology. Such spin-momentum locking property is the photonic analog of quantum spin Hall effect. Figure 5c–f show the simulation results (calculated by COMSOL Multiphysics) of optical propagation along the two interfaces, when the operation frequency of all excited source is *ω* = 0.9*ω*_op_. The topological edge state can be robust against the ‘SYSU’ shaped bending, as depicted in Figure 5c. The incident light couples to rightward wave and passes through the bending interface without backscattering, even though using a spinless *E*_z_-polarization source (black dot). For comparison, we also give a control case of gapped edge dispersion supported by an interface between two ordinary opacities (medium 1 and medium 2). Figure 5e and f have the same numerical setup to the left panel, except for topologically trivial interface. Due to lack of topological protection, the interface wave is failure against both unidirectional excitation and robust propagation.

Finally, we will discuss the possible experiment to mimic the topological interface above. Note that the bi-anisotropic metamaterials have been constructed by EM-dual ‘meta-atom’ between two metal plates [11], [55]. Similarly, we show the concrete designs to achieve the topologically non-trivial and trivial opacities in microwave regime [with the operation frequency from 2.0 to 2.5 GHz], which maintain EM duality and non-zero bianisotropy (see Appendix F of the Supplementary Materials). We have designed two types of meta-atoms in millimeter scale, where one of them is denoted by gyro-scope atom and the other is close-ring atom, to be homogenized as EM-dual PCMs. The analytic derivations prove that the first-order waveguide modes in EM-dual medium sandwiched by two perfect-electric-conductor (PEC) plates can render the strong effective bianisotropy *ξ*_*eff*_. When these two types of meta-atoms are placed inside the 38-mm-width PEC waveguides, the close-ring-atom waveguide serves as topologically non-trivial opacity due to its effective topological index $\gamma_{\text{eff}}={\left(\varepsilon_{p}/\sqrt{\rho }\right)}^{2}-\xi_{\text{eff}}^{2}<0$ , while the gyro-scope-atom waveguide atom serves as trivial opacity since the operation frequency from 2.0 to 2.5 GHz is below cut-off frequency of first-order modes of gyro-scope atom. Therefore, we have successfully designed two types of meta-atoms in PEC waveguide to mimic the topological interface in Figure 5.

## 3 Conclusions

In summary, we have successfully applied a topological index based on EM parameters to demonstrate the topologically nontrivial properties in a class of 2D PCM with bi-anisotropic response. With an analytic deduction, we have concluded that the topology is determined by the sign of topological index. An example with Lorentz-like dispersion is given to verify this key point, including the evolution of topological index, pseudo surface impedance of bulk-edge correspondence, and topologically edge states. Since such index only depends on EM parameters, the proposal of EM-parameter method will benefit to simplify the design of topological metamaterial in some manner, and will lead to novel fundamental physics and device applications in the field of metamaterial.

## Supplementary Material

Supplementary Material Details

## References

[1] Hasan M., Kane C. (2010). Colloquium: topological insulators. *Rev. Mod. Phys.*.

[2] Qi X.-L., Zhang S.-C. (2011). Topological insulators and superconductors. *Rev. Mod. Phys.*.

[3] Lu L., Joannopoulos J. D., Soljacic M. (2014). Topological photonics. *Nat. Photon.*.

[4] Khanikaev A. B., Shvets G. (2017). Two-dimensional topological photonics. *Nat. Photon.*.

[5] Ozawa T. (2019). Topological photonics. *Rev. Mod. Phys.*.

[6] Kim M., Jacob Z., Rho J. (2020). Recent advances in 2D, 3D and higher-order topological photonics. *Light: Sci. Appl.*.

[7] Tang G. J., He X. T., Shi F. L., Liu J. W., Chen X. D., Dong J. W. (2022). Topological photonic crystals: physics, designs, and applications. *Laser Photon. Rev.*.

[8] Haldane F., Raghu S. (2008). Possible realization of directional optical waveguides in photonic crystals with broken time-reversal symmetry. *Phys. Rev. Lett.*.

[9] Wang Z., Chong Y., Joannopoulos J. D., Soljacic M. (2009). Observation of unidirectional backscattering-immune topological electromagnetic states. *Nature*.

[10] Khanikaev A. B., Hossein Mousavi S., Tse W.-K., Kargarian M., MacDonald A. H., Shvets G. (2013). Photonic topological insulators. *Nat. Mater.*.

[11] Chen W.-J. (2014). Experimental realization of photonic topological insulator in a uniaxial metacrystal waveguide. *Nat. Commun.*.

[12] He C. (2016). Photonic topological insulator with broken time-reversal symmetry. *Proc. Natl. Acad. Sci.*.

[13] Hafezi M., Mittal S., Fan J., Migdall A., Taylor J. M. (2013). Imaging topological edge states in silicon photonics. *Nat. Photon.*.

[14] Wu L.-H., Hu X. (2015). Scheme for achieving a topological photonic crystal by using dielectric material. *Phys. Rev. Lett.*.

[15] Barik S. (2018). A topological quantum optics interface. *Science*.

[16] Barik S., Miyake H., DeGottardi W., Waks E., Hafezi M. (2016). Two-dimensionally confined topological edge states in photonic crystals. *New J. Phys.*.

[17] Ma T., Shvets G. (2016). All-Si valley-Hall photonic topological insulator. *New J. Phys.*.

[18] Dong J.-W., Chen X.-D., Zhu H., Wang Y., Zhang X. (2017). Valley photonic crystals for control of spin and topology. *Nat. Mater.*.

[19] Chen X.-D., Zhao F.-L., Chen M., Dong J.-W. (2017). Valley-contrasting physics in all-dielectric photonic crystals: orbital angular momentum and topological propagation. *Phys. Rev. B*.

[20] Shalaev M. I., Walasik W., Tsukernik A., Xu Y., Litchinitser N. M. (2019). Robust topologically protected transport in photonic crystals at telecommunication wavelengths. *Nat. Nanotechnol.*.

[21] He X.-T. (2019). A silicon-on-insulator slab for topological valley transport. *Nat. Commun.*.

[22] Xiao M., Zhang Z. Q., Chan C. T. (2014). Surface impedance and bulk band geometric phases in one-dimensional systems. *Phys. Rev. X*.

[23] Liu F., Wakabayashi K. (2017). Novel topological phase with a zero berry curvature. *Phys. Rev. Lett.*.

[24] Xie B.-Y. (2018). Second-order photonic topological insulator with corner states. *Phys. Rev. B*.

[25] Xie B.-Y. (2019). Visualization of higher-order topological insulating phases in two-dimensional dielectric photonic crystals. *Phys. Rev. Lett.*.

[26] Chen X.-D., Deng W.-M., Shi F.-L., Zhao F.-L., Chen M., Dong J.-W. (2019). Direct observation of corner States in second-order topological photonic crystal slabs. *Phys. Rev. Lett.*.

[27] El Hassan A., Kunst F. K., Moritz A., Andler G., Bergholtz E. J., Bourennane M. (2019). Corner states of light in photonic waveguides. *Nat. Photon.*.

[28] Ota Y. (2019). Photonic crystal nanocavity based on a topological corner state. *Optica*.

[29] Noh J. (2018). Topological protection of photonic mid-gap defect modes. *Nat. Photon.*.

[30] Peterson C. W., Benalcazar W. A., Hughes T. L., Bahl G. (2018). A quantized microwave quadrupole insulator with topologically protected corner states. *Nature*.

[31] Serra-Garcia M. (2018). Observation of a phononic quadrupole topological insulator. *Nature*.

[32] Harari G. (2018). Topological insulator laser: theory. *Science*.

[33] Bandres M. A. (2018). Topological insulator laser: experiments. *Science*.

[34] Blanco-Redondo A., Bell B., Oren D., Eggleton B. J., Segev M. (2018). Topological protection of biphoton states. *Science*.

[35] Mittal S., Goldschmidt E. A., Hafezi M. (2018). A topological source of quantum light. *Nature*.

[36] Bansil A., Lin H., Das T. (2016). Colloquium: topological band theory. *Rev. Mod. Phys.*.

[37] Joannopoulos J. D., Johnson S. G., Winn J. N., Meade R. D. (2011). *Photonic Crystals: Molding the Flow of Light*.

[38] Soukoulis C. M., Wegener M. (2011). Past achievements and future challenges in the development of three-dimensional photonic metamaterials. *Nat. Photon.*.

[39] Jahani S., Jacob Z. (2016). All-dielectric metamaterials. *Nat. Nanotech.*.

[40] Yu Z., Veronis G., Wang Z., Fan S. (2008). One-Way electromagnetic waveguide formed at the interface between a plasmonic metal under a static magnetic field and a photonic crystal. *Phys. Rev. Lett.*.

[41] Davoyan A., Engheta N. (2013). Theory of wave propagation in magnetized near-zero-epsilon metamaterials: evidence for one-way photonic States and magnetically switched transparency and opacity. *Phys. Rev. Lett.*.

[42] Silveirinha M. G. (2015). Chern invariants for continuous media. *Phys. Rev. B*.

[43] Silveirinha M. G. P·T·Dsymmetry-protected scattering anomaly in optics. *Phys. Rev. B*.

[44] Gao W. (2015). Topological photonic phase in chiral hyperbolic metamaterials. *Phys. Rev. Lett.*.

[45] Xiao M., Lin Q., Fan S. (2016). Hyperbolic Weyl point in reciprocal chiral metamaterial. *Phys. Rev. Lett.*.

[46] Zangeneh-Nejad F., Fleury R. (2020). Zero-Index weyl metamaterials. *Phys. Rev. Lett.*.

[47] Yang B. (2018). Ideal Weyl points and helicoid surface states in artificial photonic crystal structures. *Science*.

[48] Ma S. (2021). Linked Weyl surfaces and Weyl arcs in photonic metamaterials. *Science*.

[49] He C., Zhao L., Zhang S., Zhou L., Ma S. (2025). Intrinsic topological Hinge states induced by boundary gauge fields in photonic metamaterials. *eLight*.

[50] Horsley S. A. R., Woolley M. (2021). Zero-refractive-index materials and topological photonics. *Nat. Phys.*.

[51] Zhang R.-Y. (2025). Bulk–spatiotemporal vortex correspondence in gyromagnetic zero-index media. *Nature*.

[52] Lannebère S., Silveirinha M. G. (2019). Photonic analogues of the Haldane and Kane-Mele models. *Nanophotonics*.

[53] Lustig E. (2019). Photonic topological insulator in synthetic dimensions. *Nature*.

[54] Yu D. Comprehensive review on developments of synthetic dimensions. *Photon. Insights*.

[55] Slobozhanyuk A. P., Khanikaev A. B., Filonov D. S., Smirnova D. A., Miroshnichenko A. E., Kivshar Y. S. (2016). Experimental demonstration of topological effects in bianisotropic metamaterials. *Sci. Rep.*.

