# Supplementary material for: Classification of quantum-spin-hall topological phase in 2D photonic continuous media using electromagnetic parameters
Source: Nanophotonics. 2025 Oct 23;14(27):5385–93. doi: 10.1515/nanoph-2025-0451 (PMC12717913; doi:10.1515/nanoph-2025-0451)
Supplement: Supplementary file 1 — Supplementary Material Details [file j_nanoph-2025-0451_suppl_001.pdf]

Supplementary Material for Classification of Quantum-Spin-Hall Topological Phase in 2D Photonic Continuous Media Using Electromagnetic Parameters

Xin-Tao He, Shuo-Shi Zhang, Xiao-Dong Chen and Jian-Wen Dong*

School of Physics & State Key Laboratory of Optoelectronic Materials and Technologies,
Sun Yat-sen University, Guangzhou 510275, China
* Email: dongjwen@mail.sysu.edu.cn

This file includes:

Appendix A: Construct of pseudo spin in photonic continuous medium

Appendix B: Effective Hamiltonian in 2D photonic system

Appendix C: Topological properties of PCM in momentum space

Appendix D: Nonlocal approximation for Lorentz-like dispersion

Appendix E: Master equation correspondence between MO and BI media

Appendix F: Proposal of EM-dual PCMs in microwave regime

Appendix A: Construct of pseudo spin in photonic continuous medium

Consider a photonic continuous medium (PCM) with bi-anisotropic (BI) response, its electromagnetic (EM) parameters have the following form,

$$\overleftrightarrow{\varepsilon}_r = \begin{pmatrix} \varepsilon_p & 0 & 0 \\ 0 & \varepsilon_p & 0 \\ 0 & 0 & \varepsilon_z \end{pmatrix},\ \overleftrightarrow{\mu}_r = \begin{pmatrix} \mu_p & 0 & 0 \\ 0 & \mu_p & 0 \\ 0 & 0 & \mu_z \end{pmatrix},\ \overleftrightarrow{\chi} = \begin{pmatrix} 0 & -i\xi & \\ i\xi & 0 & \\ & & 0 \end{pmatrix}, \tag{S1}$$

where $\overleftrightarrow{\varepsilon}_r$, $\overleftrightarrow{\mu}_r$ and $\overleftrightarrow{\chi}$ are the relative permittivity tensor, relative permeability tensor and bi-anisotropic tensor, respectively. The constructive relations can be written as,

$$\begin{aligned} \vec{D} &= \varepsilon_0 \overleftrightarrow{\varepsilon}_r \vec{E} + \left(\overleftrightarrow{\chi}/c\right)\vec{H}, \\ \vec{B} &= \mu_0 \overleftrightarrow{\mu}_r \vec{H} + \left(\overleftrightarrow{\chi}/c\right)\vec{E}. \end{aligned} \tag{S2}$$

For harmonic waves in passive system, the Maxwell equations with bi-anisotropic response can be rewritten as,

$$\nabla \times \vec{E} = i\omega\left[\mu_0 \overleftrightarrow{\mu}_r \vec{H} + \left(\overleftrightarrow{\chi}/c\right)\vec{E}\right], \tag{S3}$$

$$\nabla \times \vec{H} = -i\omega\left[\varepsilon_0 \overleftrightarrow{\varepsilon}_r \vec{E} + \left(\overleftrightarrow{\chi}/c\right)\vec{H}\right], \tag{S4}$$

where ε_0, μ_0 and c are the permittivity, permeability and velocity of light in vacuum, respectively. In general, different optical materials respond differently for electric field and magnetic field i.e., EM-dual symmetry breaking. For example, the ratio of medium 1 ($\rho_1 = \varepsilon_{p1}/\mu_{p1} = \varepsilon_{z1}/\mu_{z1}$) is quite different to that of medium 2 ($\rho_2 = \varepsilon_{p2}/\mu_{p2} = \varepsilon_{z2}/\mu_{z2}$). In order to construct a pair of pseudo spin degenerating, one of the solutions is to retrieve electromagnetic-dual parameter that ensure the ratio ρ as a constant in the whole space. In this way, the Maxwell equations can be divided into two non-relativistic equations for two sets of decoupled vectors,

$$\begin{pmatrix} 0 & 0 & i\frac{\partial}{\partial y} \\ 0 & 0 & -i\frac{\partial}{\partial x} \\ i\frac{\partial}{\partial y} & -i\frac{\partial}{\partial x} & 0 \end{pmatrix} \begin{pmatrix} \sqrt{\varepsilon_0\rho}E_x - \sqrt{\mu_0}H_x \\ \sqrt{\varepsilon_0\rho}E_y - \sqrt{\mu_0}H_y \\ \sqrt{\varepsilon_0\rho}E_z + \sqrt{\mu_0}H_z \end{pmatrix} = \frac{\omega}{c} \begin{pmatrix} \varepsilon_p/\sqrt{\rho} & i\xi & 0 \\ -i\xi & \varepsilon_p/\sqrt{\rho} & 0 \\ 0 & 0 & \varepsilon_z/\sqrt{\rho} \end{pmatrix} \begin{pmatrix} \sqrt{\varepsilon_0\rho}E_x - \sqrt{\mu_0}H_x \\ \sqrt{\varepsilon_0\rho}E_y - \sqrt{\mu_0}H_y \\ \sqrt{\varepsilon_0\rho}E_z + \sqrt{\mu_0}H_z \end{pmatrix}, \quad \text{(S5)}$$

$$\begin{pmatrix} 0 & 0 & -i\frac{\partial}{\partial y} \\ 0 & 0 & i\frac{\partial}{\partial x} \\ -i\frac{\partial}{\partial y} & i\frac{\partial}{\partial x} & 0 \end{pmatrix} \begin{pmatrix} \sqrt{\varepsilon_0\rho}E_x + \sqrt{\mu_0}H_x \\ \sqrt{\varepsilon_0\rho}E_y + \sqrt{\mu_0}H_y \\ \sqrt{\varepsilon_0\rho}E_z - \sqrt{\mu_0}H_z \end{pmatrix} = \frac{\omega}{c} \begin{pmatrix} \varepsilon_p/\sqrt{\rho} & -i\xi & 0 \\ i\xi & \varepsilon_p/\sqrt{\rho} & 0 \\ 0 & 0 & \varepsilon_z/\sqrt{\rho} \end{pmatrix} \begin{pmatrix} \sqrt{\varepsilon_0\rho}E_x + \sqrt{\mu_0}H_x \\ \sqrt{\varepsilon_0\rho}E_y + \sqrt{\mu_0}H_y \\ \sqrt{\varepsilon_0\rho}E_z - \sqrt{\mu_0}H_z \end{pmatrix}. \quad \text{(S6)}$$

Such two sets of decoupled vectors are a pair of pseudospin-polarized states as time-reversal partner, i.e. $\vec{\psi}^+ = \left(\sqrt{\varepsilon_0\rho}E_x - \sqrt{\mu_0\rho}H_x \quad \sqrt{\varepsilon_0\rho}E_y - \sqrt{\mu_0}H_y \quad \sqrt{\varepsilon_0\rho}E_z + \sqrt{\mu_0}H_z\right)^T$ for pseudospin up (in-phase of electric and magnetic fields in z component) and $\vec{\psi}^- = \left(\sqrt{\varepsilon_0\rho}E_x + \sqrt{\mu_0}H_x \quad \sqrt{\varepsilon_0\rho}E_y + \sqrt{\mu_0}H_y \quad \sqrt{\varepsilon_0\rho}E_z - \sqrt{\mu_0}H_z\right)^T$ for pseudospin down (antiphase of electric and magnetic fields in z component).

Appendix B: Effective Hamiltonian in 2D photonic system

Next, we will discuss a special case of EM duality with $\rho = 1$ [$\overleftrightarrow{\varepsilon}_r = \overleftrightarrow{\mu}_r = diag\left(\varepsilon_p, \varepsilon_p, \varepsilon_z\right)$], and thus the master equation can be simplified as,

$$\begin{pmatrix} 0 & 0 & \pm i\frac{\partial}{\partial y} \\ 0 & 0 & \mp i\frac{\partial}{\partial x} \\ \pm i\frac{\partial}{\partial y} & \mp i\frac{\partial}{\partial x} & 0 \end{pmatrix} \begin{pmatrix} \sqrt{\varepsilon_0}E_x \mp \sqrt{\mu_0}H_x \\ \sqrt{\varepsilon_0}E_y \mp \sqrt{\mu_0}H_y \\ \sqrt{\varepsilon_0}E_z \pm \sqrt{\mu_0}H_z \end{pmatrix} = \frac{\omega}{c} \begin{pmatrix} \varepsilon_p & \pm i\xi & 0 \\ \mp i\xi & \varepsilon_p & 0 \\ 0 & 0 & \varepsilon_z \end{pmatrix} \begin{pmatrix} \sqrt{\varepsilon_0}E_x \mp \sqrt{\mu_0}H_x \\ \sqrt{\varepsilon_0}E_y \mp \sqrt{\mu_0}H_y \\ \sqrt{\varepsilon_0}E_z \pm \sqrt{\mu_0}H_z \end{pmatrix}. \quad \text{(S7)}$$

We define the differential operator $\overleftrightarrow{N}^{\pm}$ and material matrix $\overleftrightarrow{M}^{\pm}$ as follow,

$$\overleftrightarrow{N}^{\pm}=\pm\begin{pmatrix} 0 & 0 & i\dfrac{\partial}{\partial y} \\ 0 & 0 & -i\dfrac{\partial}{\partial x} \\ i\dfrac{\partial}{\partial y} & -i\dfrac{\partial}{\partial x} & 0 \end{pmatrix},\ \overleftrightarrow{M}^{\pm}=\begin{pmatrix} \varepsilon_p & \pm i\xi & 0 \\ \mp i\xi & \varepsilon_p & 0 \\ 0 & 0 & \varepsilon_z \end{pmatrix}, \tag{S8}$$

where the superscripts + and – represent pseudospin-up and -down cases, respectively. We also have the inverse of material matrix and its elements,

$$\left(\overleftrightarrow{M}^{\pm}\right)^{-1}=\begin{pmatrix} \alpha_p & \mp i\alpha_k & 0 \\ \pm i\alpha_k & \alpha_p & 0 \\ 0 & 0 & \alpha_z \end{pmatrix},\ \alpha_p=\frac{\varepsilon_p}{\varepsilon_p^2-\xi^2},\alpha_k=\frac{\xi}{\varepsilon_p^2-\xi^2},\alpha_z=\frac{1}{\varepsilon_z}. \tag{S9}$$

With a simple deduction, the Maxwell equations based on Eq. (S7) can be generalized to Schrödinger-like formation,

$$\left(\overleftrightarrow{M}^{\pm}\right)^{-1}\overleftrightarrow{N}^{\pm}\vec{\psi}^{\pm}=\overleftrightarrow{H}^{\pm}\vec{\psi}^{\pm}=\frac{\omega}{c}\vec{\psi}^{\pm}. \tag{S10}$$

$\overleftrightarrow{H}^{\pm}$ is the characteristic matrix based on Maxwell equations. Note that it is similar to the role of Hamiltonian in quantum system, and can be called the effective Hamiltonian. For simplicity, the eigenfields are assumed to be plane-wave form as $\vec{\psi}^{\pm}=\vec{\varphi}^{\pm}\exp\left(i\vec{k}\bullet\vec{r}\right)$, where $\vec{\varphi}^{\pm}$ is the complex amplitude of the pseudospin-polarized states. By means of the eigenvalues of effective Hamiltonian, the bulk dispersion is given,

$$k_x^2+k_y^2=k^2=\left(\frac{\omega}{c}n_{eff}\right)^2, \tag{S11}$$

where $n_{eff}=\sqrt{\varepsilon_z\left(\varepsilon_p^2-\xi^2\right)/\varepsilon_p}$ acts as the effective refractive index. This is the characteristic equation for both pseudospin-up and –down modes, as they degenerate simultaneously in the bulk. Furthermore, the amplitudes of eigenmodes can be analytically written as,

$$\vec{\varphi}^{\pm} = \begin{pmatrix} -ik_x\alpha_k / k_0 \mp k_y\alpha_p / k_0 \\ \pm k_x\alpha_p / k_0 - ik_y\alpha_k / k_0 \\ 1 \end{pmatrix}, \tag{S12}$$

where $k_0 = \omega/c$ is the wave number in vacuum.

Appendix C: Topological properties of PCM in momentum space

To study the topological properties of those bulk bands and gaps, we should attain the Berry information, including the Berry connection $\vec{A}^{\pm}$, Berry curvature $\Omega^{\pm}$ and the corresponding spin Chern number C_s. Here, Berry connection $\vec{A}^{\pm}$ is the synthetic gauge-dependent field, while Berry curvature $\Omega^{\pm} = \nabla_k \times \vec{A}^{\pm}$ is a gauge-independent function and represents the flux of Berry gauge field. For PCM, there are two issues in the retrieval of spin Chern number. For one thing, the effective Hamiltonian should be Hermitian formalism so as to provide suitable inner product for the computation of Berry connection [1]. In this paper, we concern about dispersive (frequency-dependent) PCMs, such that Eq. (S10) will be not straightforward for a Hermitian formulation. This issue for dispersive media can be recovered by a generalized method.

We are dealing with a Hermitian eigenvalue problem when the elements of material matrix $\vec{\overleftrightarrow{M}}^{\pm}$ are independent of frequency ω. However, what we concern about in this paper are homogeneous dispersive (frequency-dependent) materials, such that Eq. (S9) will be not straightforward for a Hermitian formulation. This issue for dispersive media can be recovered by a generalized method [2]. In this way, the Berry connection $\vec{A}^{\pm}$ associated with the generalized problem for 2D system are obtained as follow,

$$\vec{A}^{\pm} = \frac{\mathrm{Re}\left\{ i\left(\vec{\varphi}^{\pm}\right)^{*} \cdot \frac{\partial}{\partial\omega}\left[\omega \overleftrightarrow{M}^{\pm}\right] \cdot \nabla_{k} \vec{\varphi}^{\pm} \right\}}{\left(\vec{\varphi}^{\pm}\right)^{*} \cdot \frac{\partial}{\partial\omega}\left[\omega \overleftrightarrow{M}^{\pm}\right] \cdot \vec{\varphi}^{\pm}} = A_{x}^{\pm}\vec{k}_{x} + A_{y}^{\pm}\vec{k}_{y}\,, \tag{S13}$$

where $\nabla_{k} = \frac{\partial}{\partial k_{x}}\vec{k}_{x} + \frac{\partial}{\partial k_{y}}\vec{k}_{y}$ is the Laplace operator in 2D momentum space. The Berry connection is a gauge-dependent field, while the associated gauge-independent function is Berry curvature $\Omega^{\pm}$,

$$\Omega^{\pm} = \nabla_{k} \times \vec{A}^{\pm} = \frac{\partial A_{y}^{\pm}}{\partial k_{x}} - \frac{\partial A_{x}^{\pm}}{\partial k_{y}}\,, \tag{S14}$$

By the integral of the Berry curvatures over entire k space, we have a quantized invariant to define the topology of pseudospin-up and –down channels,

$$C^{\pm} = \frac{1}{2\pi}\iint \Omega^{\pm} dk_{x} dk_{y}\,, \tag{S15}$$

which is called the Chern number. Owing to time-reversal invariance, the net Chern number always vanishes, i.e. $C^{+} + C^{-} = 0$. However, we still have spin Chern number to characterize the topological phase of the overall system, yielding

$$C_{s} = \frac{C^{+} - C^{-}}{2} = C^{+}\,. \tag{S16}$$

This topological invariant is the photonic analog of spin Hall conductance and plays the role in the classification of topology of optical materials with TRS. When the Berry curvature is smooth in the whole momentum space, the 2D integration of Eq. (S15) satisfies Stokes' theorem that can be reduced to a line integral of Berry connection along the equifrequency contour of infinite wavevector ($k = \infty$),

$$C_{s} = C^{+} = \frac{1}{2\pi}\iint \Omega^{+} dk_{x} dk_{y} = \frac{1}{2\pi}\oint_{k=\infty} \vec{A}^{+} \cdot d\vec{l} \tag{S17}$$

which leads to trivial distribution of Berry connection ($C_s = 0$) because the optical material response of a well-behaved pseudo Hamiltonian tends to be the same as vacuum at $k = \infty$. On the other hand, the occurrence of topological nontrivial phase is derived from the singularity of Berry curvature, which indicates Eq. (S17) is valid. This issue can be recovered by adding a summation of line integrals in the vicinity of singular k points,

$$C_s = \frac{1}{2\pi}\left(-\sum_{k_i}\oint_{R^+}\vec{A}^+\cdot d\vec{l} + \oint_{k=\infty}\vec{A}^+\cdot d\vec{l}\right) \tag{S18}$$

where R^+ is a circle in the vicinity of singular $\vec{k}_i$ point. The summation determines nonzero spin Chern number, indicating that the singularity of Berry curvature makes contribution to the nontrivial case of topological phase. Consequently, it is significant to explore the deterministic condition of singular Berry curvature in bi-anisotropic metamaterial so as to guarantee the existence of topological nontrivial phase.

With a substitution of Eq. (S8) and Eq. (S12) into Eqs. (S13-S14), the Berry curvature can be deduced as the following analytic expression,

$$\Omega^\pm = \mp \frac{\left(\xi + \omega\frac{\partial \xi}{\partial \omega}\right)\left(\varepsilon_p^2 + \xi^2\right) - 2\xi\varepsilon_p\left(\varepsilon_p + \omega\frac{\partial \varepsilon_p}{\partial \omega}\right)}{\left(\varepsilon_p^2 - \xi^2\right)^2}\frac{2}{W_0 k_0^2} \tag{S19}$$

where

$$W_0 = \left(\vec{\varphi}^\pm\right)^* \cdot \frac{\partial}{\partial \omega}\left[\omega \overleftrightarrow{M}^\pm\right]\cdot\vec{\varphi}^\pm \tag{S20}$$

is proportional to the time-averaged energy density (a positive real value). The singularity means infinite value of Berry curvature at finite frequency.

Appendix D: Nonlocal approximation for Lorentz-like dispersion

For another, to ensure that the spin Chern number is an integer, the momentum space should be a closed surface without boundary (e.g. Brillouin zone in photonic crystals). But the PCM is failure to meet this criteria. To overcome this limitation and obtain a well-behaved Hamiltonian over the entire k space, one can introduce a nonlocal approximation with a high-wavenumber spatial cutoff into the material dispersion.

In this work, we consider an electromagnetic-dual ($\varepsilon = \mu$) BI medium with Lorentz-like dispersion,

$$\varepsilon_p = \mu_p = 1 + \frac{\omega_A^2}{\omega_{op}^2 - \omega^2}, \xi = \frac{\omega_B \omega}{\omega_{ok}^2 - \omega^2}, \varepsilon_z = \mu_z = 1 + \frac{\omega_C^2}{\omega_{oz}^2 - \omega^2}, \tag{S21}$$

where ω_{op}, ω_{ok} and ω_{oz} are the oscillation frequencies of material parameters, while ω_A, ω_B, ω_C represent the resonance strength, respectively. Applying these material dispersions to Eq. (11), we have analytic solution of the dispersion relation with $\omega_{ok} = \omega_{op}$, $\omega_{oz} = 0.8\omega_{op}$, $\omega_A = 1.5\omega_{op}$, $\omega_B = 0.75\omega_{op}$, $\omega_C = 0.9\omega_{op}$. As shown in the main text of Fig. 1(a), there are six characteristic frequencies, i.e. $\omega = \omega_{oz}, \omega_{op}, \sqrt{\omega_{op}^2 + \omega_A^2}$ at $k = \infty$ and $\omega = \sqrt{\omega_{oz}^2 + \omega_C^2}, \frac{-\omega_B + \sqrt{\omega_B^2 + 4\left(\omega_{op}^2 + \omega_A^2\right)}}{2}, \frac{\omega_B + \sqrt{\omega_B^2 + 4\left(\omega_{op}^2 + \omega_A^2\right)}}{2}$ at $k = 0$, that separating the bulk bands (Arabic numerals 1-4 with pink background) from each other. The cyan regions between bulk bands are relative to complete bandgaps and are labeled as Roman numerals I-III. For the band 4, the material response will be identified with those of the vacuum when $\omega \to \infty$. This property guarantees the pseudo Hamiltonian is a well-behaved operator as $k \to \infty$ of band 4 and thus ensures the retrieval of integer topological invariant. However, the situations are quite different for the low-frequency bands 1-3. So the corresponding spin Chern numbers are not integer. To overcome this limitation, one can introduce a nonlocal approximation with a high-

wavenumber spatial cutoff into the material dispersion,

$$
\begin{aligned}
\varepsilon_p = \mu_p &= 1 + \frac{1}{1 + k^2 / k_{\max}^2} \frac{\omega_A^2}{\omega_{op}^2 - \omega^2} \\
\xi &= \frac{1}{1 + k^2 / k_{\max}^2} \frac{\omega_B \omega}{\omega_{ok}^2 - \omega^2} \\
\varepsilon_z = \mu_z &= 1 + \frac{1}{1 + k^2 / k_{\max}^2} \frac{\omega_C^2}{\omega_{oz}^2 - \omega^2}
\end{aligned}
, \qquad \text{(S22)}
$$

where $k_{\max}$ is the spatial cutoff wavenumber. For $k \ll k_{\max}$, the regularized material dispersion [Eq. (S22)] is coincident with the original case [Eq. (S21)]. The different responses are only emergent for large wavenumber ($k \sim k_{\max}$ or $k > k_{\max}$). In fact, the high-k mode ($k \gg \omega / c$) is difficult to be excited in a practically photonic system. Consequently, the difference between Eq. (S21) and Eq. (S22) is only attributed to mathematical behavior rather than optical response if the spatial cutoff $k_{\max}$ is large enough. In this paper, we set $k_{\max} = 10000\omega_{op}/c$.

Appendix E: Master equation correspondence between MO and BI media

In this section, we will show the correspondence between bi-anisotropic (BI) and magneto-optical (MO) media by solving the Maxwell equations, indicating that the EM parameter method providing in this work can be developed to describe MO medium. As shown above [see Eq. (S7)], the master equations of BI media can be divided into two non-relativistic equations for two sets of decoupled vectors. Consider a PCM with gyroelectric response, whose EM parameters have the following form,

$$
\vec{\varepsilon}_r = \begin{pmatrix} \varepsilon_p & i\kappa_e & 0 \\ -i\kappa_e & \varepsilon_p & 0 \\ 0 & 0 & \varepsilon_z \end{pmatrix}, \ \vec{\mu}_r = \begin{pmatrix} \mu_p & 0 & 0 \\ 0 & \mu_p & 0 \\ 0 & 0 & \mu_z \end{pmatrix}, \qquad \text{(S23)}
$$

where κ_e is the gyroelectric coefficient as the external magnetic field along the -*z* direction. As

the MO effect of gyroelectric medium only works on TE-polarized wave (E_x, E_y, H_z), the constitutive relations for TE polarization are denoted by μ_z and $\begin{pmatrix} \varepsilon_p & i\kappa_e \\ -i\kappa_e & \varepsilon_p \end{pmatrix}$. Thus we can have the master equation of gyroelectric medium as,

$$\begin{pmatrix} 0 & 0 & i\frac{\partial}{\partial y} \\ 0 & 0 & -i\frac{\partial}{\partial x} \\ i\frac{\partial}{\partial y} & -i\frac{\partial}{\partial x} & 0 \end{pmatrix} \begin{pmatrix} \sqrt{\varepsilon_0}E_x \\ \sqrt{\varepsilon_0}E_y \\ \sqrt{\mu_0}H_z \end{pmatrix} = \frac{\omega}{c} \begin{pmatrix} \varepsilon_p & i\kappa_e & 0 \\ -i\kappa_e & \varepsilon_p & 0 \\ 0 & 0 & \mu_z \end{pmatrix} \begin{pmatrix} \sqrt{\varepsilon_0}E_x \\ \sqrt{\varepsilon_0}E_y \\ \sqrt{\mu_0}H_z \end{pmatrix}. \quad \text{(S24)}$$

Similarly, when we focus on a gyromagnetic PCM, the propagation of TM-polarized wave (H_x, H_y, E_z) are denoted by ε_z and $\begin{pmatrix} \mu_p & -i\kappa_m \\ i\kappa_m & \mu_p \end{pmatrix}$. κ_m is the gyromagnetic coefficient as the external magnetic field along the +z direction. In this way, we can have the master equation of gyromagnetic medium as,

$$\begin{pmatrix} 0 & 0 & -i\frac{\partial}{\partial y} \\ 0 & 0 & i\frac{\partial}{\partial x} \\ -i\frac{\partial}{\partial y} & i\frac{\partial}{\partial x} & 0 \end{pmatrix} \begin{pmatrix} \sqrt{\mu_0}H_x \\ \sqrt{\mu_0}H_y \\ \sqrt{\varepsilon_0}E_z \end{pmatrix} = \frac{\omega}{c} \begin{pmatrix} \mu_p & -i\kappa_m & 0 \\ i\kappa_m & \mu_p & 0 \\ 0 & 0 & \varepsilon_z \end{pmatrix} \begin{pmatrix} \sqrt{\mu_0}H_x \\ \sqrt{\mu_0}H_y \\ \sqrt{\varepsilon_0}E_z \end{pmatrix}. \quad \text{(S25)}$$

For a comparison between Eq. (S7) and Eqs. (S24-25), we find that the master equations in the MO and BI media are one-to-one correspondent. The BI system is equivalent to the superposition of two MO systems with opposite magnetic field. Therefore, the optical behaviors in both media are similar, and the methods developing in BI PCM are no doubt can be extended to MO systems, e.g. the topological index and topological phase map.

Appendix F: Proposal of EM-dual PCMs in microwave regime

In this section, we will show the concrete designs to achieve the topologically non-trivial and trivial PCMs in microwave regime, which should maintain EM duality and non-zero bianisotropy. Firstly, we will prove that the waveguide modes in EM-dual medium sandwiched by two perfect-electric-conductor (PEC) plates can render the strong effective bianisotropy. Secondly, we will design two types of meta-atoms that can be homogenized as EM-dual PCMs, and place them inside the PEC waveguides.

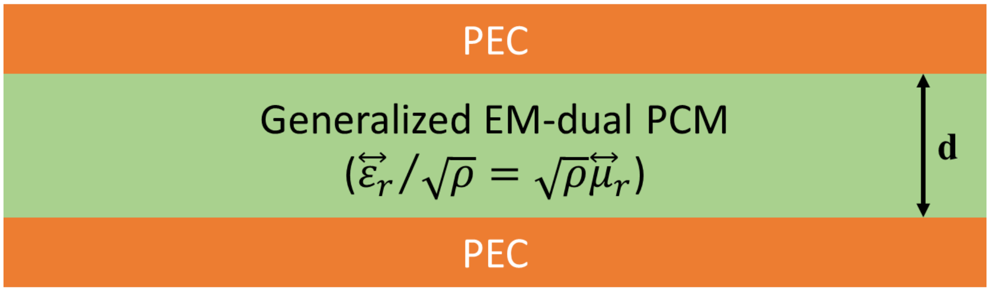

Figure S1: Schematic of EM-dual medium sandwiched by two PEC plates.

As shown in Figure S1, we consider a uniaxial medium with

$$\ddot{\varepsilon}_r/\sqrt{\rho} = \begin{pmatrix} \varepsilon_p/\sqrt{\rho} & 0 & 0 \\ 0 & \varepsilon_p/\sqrt{\rho} & 0 \\ 0 & 0 & \varepsilon_z/\sqrt{\rho} \end{pmatrix} = \sqrt{\rho}\ddot{\mu}_r = \begin{pmatrix} \sqrt{\rho}\mu_p & 0 & 0 \\ 0 & \sqrt{\rho}\mu_p & 0 \\ 0 & 0 & \sqrt{\rho}\mu_z \end{pmatrix}, \quad \text{(S26)}$$

which sandwiched by two PEC plates at $z = 0$ and $z = d$. To ensure EM duality, the ratio ρ should be a constant in the whole space. The Maxwell equations can be rewritten as,

$$\begin{pmatrix} 0 & -\frac{\partial}{\partial z} & \frac{\partial}{\partial y} \\ \frac{\partial}{\partial z} & 0 & -\frac{\partial}{\partial x} \\ -\frac{\partial}{\partial y} & \frac{\partial}{\partial x} & 0 \end{pmatrix} \begin{pmatrix} \sqrt{\varepsilon_0\rho}E_x \\ \sqrt{\varepsilon_0\rho}E_y \\ \sqrt{\varepsilon_0\rho}E_z \end{pmatrix} = i\frac{\omega}{c} \begin{pmatrix} \sqrt{\rho}\mu_p & 0 & 0 \\ 0 & \sqrt{\rho}\mu_p & 0 \\ 0 & 0 & \sqrt{\rho}\mu_z \end{pmatrix} \begin{pmatrix} \sqrt{\mu_0}H_x \\ \sqrt{\mu_0}H_y \\ \sqrt{\mu_0}H_z \end{pmatrix},$$

$$\begin{pmatrix} 0 & -\frac{\partial}{\partial z} & \frac{\partial}{\partial y} \\ \frac{\partial}{\partial z} & 0 & -\frac{\partial}{\partial x} \\ -\frac{\partial}{\partial y} & \frac{\partial}{\partial x} & 0 \end{pmatrix} \begin{pmatrix} \sqrt{\mu_0}H_x \\ \sqrt{\mu_0}H_y \\ \sqrt{\mu_0}H_z \end{pmatrix} = -i\frac{\omega}{c} \begin{pmatrix} \varepsilon_p/\sqrt{\rho} & 0 & 0 \\ 0 & \varepsilon_p/\sqrt{\rho} & 0 \\ 0 & 0 & \varepsilon_z/\sqrt{\rho} \end{pmatrix} \begin{pmatrix} \sqrt{\varepsilon_0\rho}E_x \\ \sqrt{\varepsilon_0\rho}E_y \\ \sqrt{\varepsilon_0\rho}E_z \end{pmatrix}. \tag{S27}$$

We move the term of $\frac{\partial}{\partial z}$ to the right side of equations and have,

$$\begin{pmatrix} 0 & 0 & \frac{\partial}{\partial y} \\ 0 & 0 & -\frac{\partial}{\partial x} \\ -\frac{\partial}{\partial y} & \frac{\partial}{\partial x} & 0 \end{pmatrix} \begin{pmatrix} \sqrt{\varepsilon_0\rho}E_x \\ \sqrt{\varepsilon_0\rho}E_y \\ \sqrt{\varepsilon_0\rho}E_z \end{pmatrix} = i\frac{\omega}{c} \begin{pmatrix} \varepsilon_p/\sqrt{\rho} & 0 & 0 \\ 0 & \varepsilon_p/\sqrt{\rho} & 0 \\ 0 & 0 & \varepsilon_z/\sqrt{\rho} \end{pmatrix} \begin{pmatrix} \sqrt{\mu_0}H_x \\ \sqrt{\mu_0}H_y \\ \sqrt{\mu_0}H_z \end{pmatrix} + i\frac{\omega}{c} \begin{pmatrix} 0 & -\frac{ic\partial}{\omega\partial z} & 0 \\ \frac{ic\partial}{\omega\partial z} & 0 & 0 \\ 0 & 0 & 0 \end{pmatrix} \begin{pmatrix} \sqrt{\varepsilon_0\rho}E_x \\ \sqrt{\varepsilon_0\rho}E_y \\ \sqrt{\varepsilon_0\rho}E_z \end{pmatrix},$$

$$\begin{pmatrix} 0 & 0 & \frac{\partial}{\partial y} \\ 0 & 0 & -\frac{\partial}{\partial x} \\ -\frac{\partial}{\partial y} & \frac{\partial}{\partial x} & 0 \end{pmatrix} \begin{pmatrix} \sqrt{\mu_0}H_x \\ \sqrt{\mu_0}H_y \\ \sqrt{\mu_0}H_z \end{pmatrix} = -i\frac{\omega}{c} \begin{pmatrix} \varepsilon_p/\sqrt{\rho} & 0 & 0 \\ 0 & \varepsilon_p/\sqrt{\rho} & 0 \\ 0 & 0 & \varepsilon_z/\sqrt{\rho} \end{pmatrix} \begin{pmatrix} \sqrt{\varepsilon_0\rho}E_x \\ \sqrt{\varepsilon_0\rho}E_y \\ \sqrt{\varepsilon_0\rho}E_z \end{pmatrix} - i\frac{\omega}{c} \begin{pmatrix} 0 & \frac{ic\partial}{\omega\partial z} & 0 \\ -\frac{ic\partial}{\omega\partial z} & 0 & 0 \\ 0 & 0 & 0 \end{pmatrix} \begin{pmatrix} \sqrt{\mu_0}H_x \\ \sqrt{\mu_0}H_y \\ \sqrt{\mu_0}H_z \end{pmatrix}. \tag{S28}$$

For the *m*-th-order modes of PEC waveguide, the EM field solutions have the form of

$$\begin{pmatrix} E_x \\ E_y \\ E_z \end{pmatrix} = \begin{pmatrix} e_x \sin(m\pi z/d) \\ e_y \sin(m\pi z/d) \\ -e_z \cos(m\pi z/d) \end{pmatrix}, \begin{pmatrix} H_x \\ H_y \\ H_z \end{pmatrix} = \begin{pmatrix} -h_x \cos(m\pi z/d) \\ -h_y \cos(m\pi z/d) \\ h_z \sin(m\pi z/d) \end{pmatrix}, \tag{S29}$$

where e_i and h_i are space-variant functions only in *x*-*y* plane for $i = x, y, z$. The cut-off frequency for *m*-th-order mode is $\omega_{c-m} = \frac{m\pi c}{\sqrt{\varepsilon_p\mu_z}d}$. Such form satisfies the boundary condition of PEC plate.

By substituting Eq. (S29) into Eq. (S28), the Maxwell equations can be deduced as the following expression,

$$\begin{pmatrix} 0 & 0 & \frac{\partial}{\partial y} \\ 0 & 0 & -\frac{\partial}{\partial x} \\ -\frac{\partial}{\partial y} & \frac{\partial}{\partial x} & 0 \end{pmatrix} \begin{pmatrix} \sqrt{\varepsilon_0 \rho} e_x \\ \sqrt{\varepsilon_0 \rho} e_y \\ \sqrt{\varepsilon_0 \rho} e_z \end{pmatrix} = i\frac{\omega}{c} \begin{pmatrix} \varepsilon_p / \sqrt{\rho} & 0 & 0 \\ 0 & \varepsilon_p / \sqrt{\rho} & 0 \\ 0 & 0 & \varepsilon_z / \sqrt{\rho} \end{pmatrix} \begin{pmatrix} \sqrt{\mu_0} h_x \\ \sqrt{\mu_0} h_y \\ \sqrt{\mu_0} h_z \end{pmatrix} + i\frac{\omega}{c} \begin{pmatrix} 0 & i\frac{m\pi c}{\omega d} & 0 \\ -i\frac{m\pi c}{\omega d} & 0 & 0 \\ 0 & 0 & 0 \end{pmatrix} \begin{pmatrix} \sqrt{\varepsilon_0 \rho} e_x \\ \sqrt{\varepsilon_0 \rho} e_y \\ \sqrt{\varepsilon_0 \rho} e_z \end{pmatrix},$$

$$\begin{pmatrix} 0 & 0 & \frac{\partial}{\partial y} \\ 0 & 0 & -\frac{\partial}{\partial x} \\ -\frac{\partial}{\partial y} & \frac{\partial}{\partial x} & 0 \end{pmatrix} \begin{pmatrix} \sqrt{\mu_0} h_x \\ \sqrt{\mu_0} h_y \\ \sqrt{\mu_0} h_z \end{pmatrix} = -i\frac{\omega}{c} \begin{pmatrix} \varepsilon_p / \sqrt{\rho} & 0 & 0 \\ 0 & \varepsilon_p / \sqrt{\rho} & 0 \\ 0 & 0 & \varepsilon_z / \sqrt{\rho} \end{pmatrix} \begin{pmatrix} \sqrt{\varepsilon_0 \rho} e_x \\ \sqrt{\varepsilon_0 \rho} e_y \\ \sqrt{\varepsilon_0 \rho} e_z \end{pmatrix} - i\frac{\omega}{c} \begin{pmatrix} 0 & i\frac{m\pi c}{\omega d} & 0 \\ -i\frac{m\pi c}{\omega d} & 0 & 0 \\ 0 & 0 & 0 \end{pmatrix} \begin{pmatrix} \sqrt{\mu_0} h_x \\ \sqrt{\mu_0} h_y \\ \sqrt{\mu_0} h_z \end{pmatrix} \quad \text{. (S30)}$$

In the same way as the 2D-space case in Appendix A, we can establish a pair of pseudospin-polarized states as time-reversal partner, i.e. $\vec{\varphi}^{+} = \left(\sqrt{\varepsilon_0 \rho} e_x - \sqrt{\mu_0} h_x \quad \sqrt{\varepsilon_0 \rho} e_y - \sqrt{\mu_0} h_y \quad \sqrt{\varepsilon_0 \rho} e_z + \sqrt{\mu_0} h_z \right)^T$ for pseudospin up and $\vec{\varphi}^{-} = \left(\sqrt{\varepsilon_0 \rho} e_x + \sqrt{\mu_0} h_x \quad \sqrt{\varepsilon_0 \rho} e_y + \sqrt{\mu_0} h_y \quad \sqrt{\varepsilon_0 \rho} e_z - \sqrt{\mu_0} h_z \right)^T$ for pseudospin down. Therefore, the Maxwell equations can be divided into two non-relativistic equations for these two sets of decoupled vectors,

$$\begin{pmatrix} 0 & 0 & i\frac{\partial}{\partial y} \\ 0 & 0 & -i\frac{\partial}{\partial x} \\ i\frac{\partial}{\partial y} & -i\frac{\partial}{\partial x} & 0 \end{pmatrix} \begin{pmatrix} \sqrt{\varepsilon_0 \rho} e_x - \sqrt{\mu_0} h_x \\ \sqrt{\varepsilon_0 \rho} e_y - \sqrt{\mu_0} h_y \\ \sqrt{\varepsilon_0 \rho} e_z + \sqrt{\mu_0} h_z \end{pmatrix} = \frac{\omega}{c} \begin{pmatrix} \varepsilon_p / \sqrt{\rho} & i\frac{m\pi c}{\omega d} & 0 \\ -i\frac{m\pi c}{\omega d} & \varepsilon_p / \sqrt{\rho} & 0 \\ 0 & 0 & \varepsilon_z / \sqrt{\rho} \end{pmatrix} \begin{pmatrix} \sqrt{\varepsilon_0 \rho} e_x - \sqrt{\mu_0} h_x \\ \sqrt{\varepsilon_0 \rho} e_y - \sqrt{\mu_0} h_y \\ \sqrt{\varepsilon_0 \rho} e_z + \sqrt{\mu_0} h_z \end{pmatrix}, \quad \text{(S31)}$$

$$\begin{pmatrix} 0 & 0 & -i\frac{\partial}{\partial y} \\ 0 & 0 & i\frac{\partial}{\partial x} \\ -i\frac{\partial}{\partial y} & i\frac{\partial}{\partial x} & 0 \end{pmatrix} \begin{pmatrix} \sqrt{\varepsilon_0 \rho} e_x + \sqrt{\mu_0} h_x \\ \sqrt{\varepsilon_0 \rho} e_y + \sqrt{\mu_0} h_y \\ \sqrt{\varepsilon_0 \rho} e_z - \sqrt{\mu_0} h_z \end{pmatrix} = \frac{\omega}{c} \begin{pmatrix} \varepsilon_p / \sqrt{\rho} & -i\frac{m\pi c}{\omega d} & 0 \\ i\frac{m\pi c}{\omega d} & \varepsilon_p / \sqrt{\rho} & 0 \\ 0 & 0 & \varepsilon_z / \sqrt{\rho} \end{pmatrix} \begin{pmatrix} \sqrt{\varepsilon_0 \rho} e_x + \sqrt{\mu_0} h_x \\ \sqrt{\varepsilon_0 \rho} e_y + \sqrt{\mu_0} h_y \\ \sqrt{\varepsilon_0 \rho} e_z - \sqrt{\mu_0} h_z \end{pmatrix}. \quad \text{(S32)}$$

Compared with Eqs. (S6) and (S7), it is straightforward to conclude that the PEC waveguide mode renders an effective non-zero bianisotropy $\xi_{eff} = \frac{m\pi c}{\omega d}$ for $m \neq 0$. For example, consider $m = 1$

and the operation frequency $\omega = \eta_\omega \omega_{c-1} = \eta_\omega \frac{\pi c}{d\sqrt{\varepsilon_p \mu_z}} = \eta_\omega \frac{\pi c}{d\sqrt{\varepsilon_p \varepsilon_z / \rho}}$ where $\eta_\omega > 1$, we can excite the 1st-order modes to achieve effective bianisotropy $\xi_{eff} = \frac{\pi c}{\omega d} = \eta_\omega \sqrt{\varepsilon_p \varepsilon_z / \rho}$. As presented in the main text, to realize topologically non-trivial opacity, we should have

$$\left| \frac{\xi_{eff}}{\varepsilon_p / \sqrt{\rho}} \right| = \eta_\omega \sqrt{\frac{\varepsilon_z}{\varepsilon_p}} > 1 . \tag{S33}$$

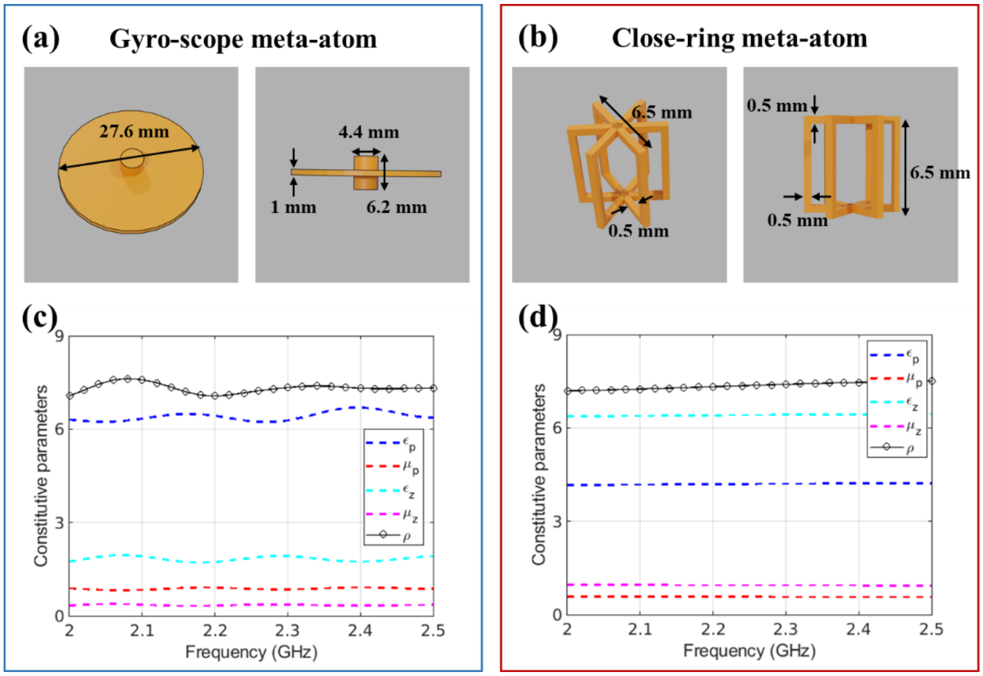

Figure S2: Design of two types of meta-atoms in mm scale to mimic non-trivial and trivial opacities. (a) and (b) Schematics of gyro-scope and close-ring meta-atoms to show the structural parameters. (c) and (d) The retrieved effective constitutive parameters of both meta-atoms.

Next, we will show the designs of two types of meta-atoms in millimeter (mm) scale, where one of them is denoted by gyro-scope (GS) meta-atom to mimic trivial opacity, and the other is close-ring (CR) meta-atom to mimic non-trivial opacity. Both two atoms are constructed by PEC in glass background and their structural parameters are shown in the schematics of Figures S2(a) and S2(b). By using standard S-parameter method[3], the effective permittivity and permeability of

each atom have been extracted, as shown in Figures S2(c) and S2(d). We can observe that the retrieved EM parameters (dashed curves) are weakly dispersive in the frequency from 2.0 to 2.5 GHz. Furthermore, the ratios ρ (black solid curves with circle markers) for both two meta-atoms are roughly equal to each other, meaning the approximation of EM-dual symmetry. In Figure S2(d), the ε_z (cyan dashed curve) of CR atom is drastically larger than ε_p (blue dashed curve), showing potential to meet the criteria of non-trivial opacity of Eq. (S33) [will be examined in the next paragraph]. Consequently, these two meta-atoms well-designed to mimic EM-dual PCMs.

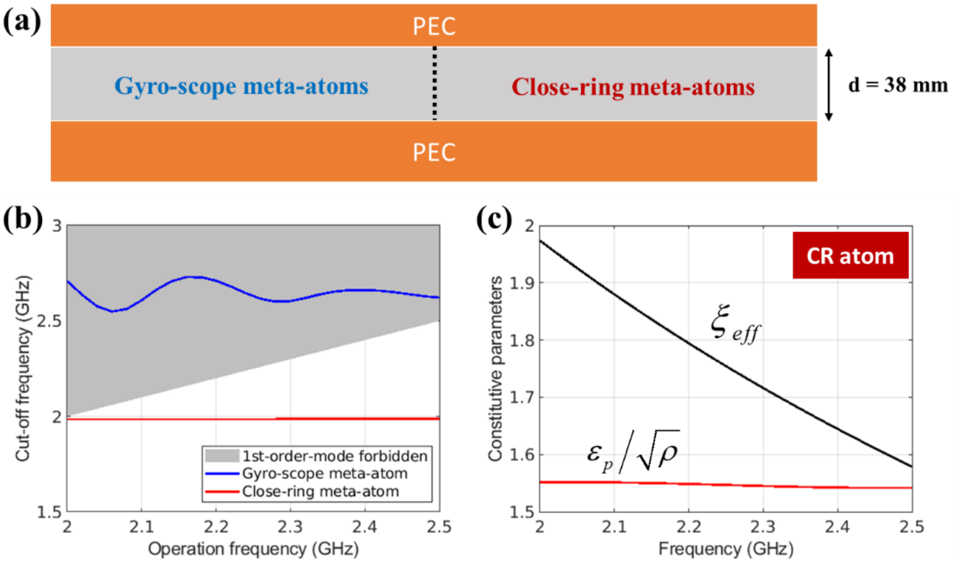

Figure S3: (a) Proposal of the topological interface in a 38-mm-width PEC waveguide, which imbeds GS meta-atoms at the left side and CR meta-atoms at the right side. (b) Cut-off frequency of the 1st-order modes as a function of operation frequency for GS atoms (blue curve) and CR atoms (red curve). (c) In-plane EM parameter and effective bianisotropy of CR atoms for 1st-order PEC waveguide mode.

Finally, we imbed these two types of meta-atoms into a PEC waveguide to form an interface (black dashed curve in Figure S3(a) and will examine their topological properties. The width of PEC waveguide is chosen to be 38 mm, to ensure the existence of 1st-order (m = 1) mode for CR atoms and the suppression of m = 1 mode for GS atoms. Figure S3(b) calculates the cut-off frequency of the m = 1 modes of PEC filling with GS atoms (blue curve) and CR atoms (red curve).

For the GS-atom waveguide, since the operation frequency from 2.0 to 2.5 GHz is below cut-off frequency, such waveguide only supports the m = 0 mode with zero effective anisotropy. Consider a z-odd-profile pseudospin source, the m = 0 mode of GS-atom waveguide can't be excited and there is lack of propagating waves inside the bulk medium, indicating the GS atom serves as trivial opacity in this case. On the other hand, the CR-atom waveguide in the operation frequency from 2.0 to 2.5 GHz can support m = 1 mode. Figure S3(c) gives the effective bianisotropy ξ_{eff} of m = 1 mode (black curve) and generalized in-plane permittivity $\varepsilon_p/\sqrt{\rho}$ (red curve). Based on the phase map in Figure 3, we can conclude that the CR-atom waveguide serves as topologically non-trivial opacity due to $\left(\varepsilon_p/\sqrt{\rho}\right)^2 - \xi_{eff}^2 < 0$ in the operation frequency from 2.0 to 2.5 GHz. Therefore, we have successfully designed two types of meta-atoms in PEC waveguide to mimic the topological interface in Figure 5 of main text.

References

[1] A. Raman and S. Fan, Physical Review Letters **2010**, 104.
[2] M. G. Silveirinha, Physical Review B **2015**, 92.
[3] D. R. Smith, S. Schultz, P. Markoš and C. M. Soukoulis, Physical Review B **2002**, 65.
